# An Elegant Approach for Complete Revascularization of the Circumflex Territory

**DOI:** 10.3390/reports9020134

**Published:** 2026-04-27

**Authors:** Ziyad Gunga, Mario Verdugo-Merchese, Matthias Kirsch, René Prêtre

**Affiliations:** Department of Cardiovascular Surgery, Lausanne University Hospital, 1011 Lausanne, Switzerlandrene.pretre@chuv.ch (R.P.)

**Keywords:** circumflex artery, total arterial revascularization, left main, CABG, new technique

## Abstract

**Background and Clinical Significance**: Revascularization of the circumflex territory remains technically challenging because of its anatomical position and the frequent need for distal branch grafting. **Case presentation:** We report the case of a 76-year-old man in whom the proximal circumflex trunk was used as the target for an in situ right internal thoracic artery routed through the transverse sinus during combined coronary and ascending aortic surgery. This approach allowed antegrade perfusion of the circumflex territory while avoiding multiple distal anastomoses. In this selected anatomical setting, the technique proved feasible and was associated with excellent intraoperative flow and 1-year radiological patency. **Conclusions:** Direct grafting of the circumflex trunk is not a new concept, but this case revisits it using a contemporary total arterial revascularization strategy. This approach may represent a useful adjunctive option in carefully selected patients with favorable circumflex anatomy.

## 1. Introduction and Clinical Significance

In left main coronary artery stenosis, it is not uncommon for the LAD and the circumflex territories to remain free from additional lesions. Revascularization typically involves arterial bypasses to both territories. While the left internal thoracic artery (LITA) is often easily grafted to the proximal LAD, revascularizing the circumflex territory presents greater challenges. The flow through these anastomoses on small arteries is much reduced compared to what could be obtained if the anastomosis was performed on the trunk of the vessel. In a previous article, Prêtre et al. described the advantages of a surgical angioplasty of the left main coronary artery over the creation of multiple distal anastomoses [1]. This elegant approach is suitable for proximal stenosis of the trunk. When its distal part is involved a more conventional revascularization is recommended. Building on this rationale, we report a case in which the proximal circumflex trunk itself was used as the direct target for an in situ RITA passed through the transverse sinus. Our intention is not to present circumflex trunk grafting as an entirely new concept, but rather to revisit and modernize it as a practical arterial solution in a carefully selected patient with left main disease and favorable circumflex anatomy.

## 2. Case Presentation

**Figure 1:**
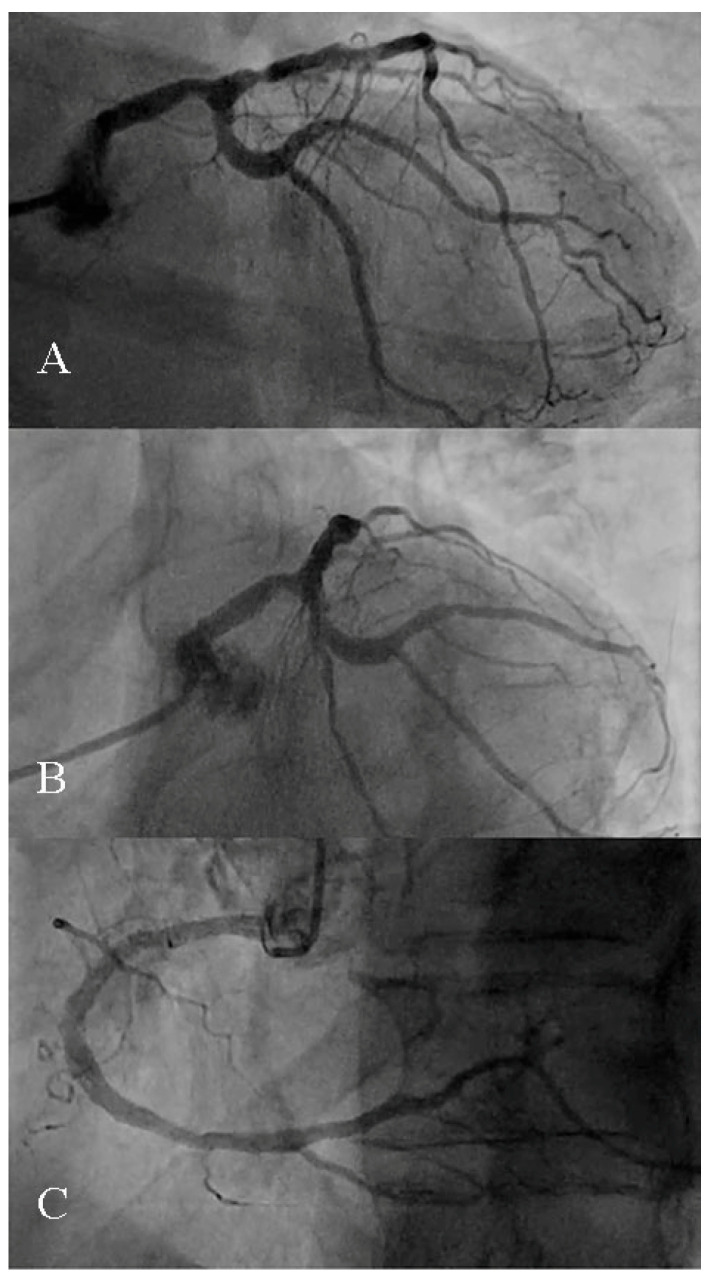
Coronary angiography displaying a stenosis on the distal left main and another one on the proximal LAD. The circumflex artery shows a trunk and two marginal arteries free of any stenosis (**A**,**B**). The right coronary artery shows no relevant stenosis (**C**).

A 76-year-old man was referred for surgical treatment of an ascending aortic aneurysm. His medical history included prior stenting of the proximal LAD. Preoperative imaging revealed significant stenosis of the distal left main trunk and at the stent site, while the circumflex artery demonstrated a short trunk before the origin of the first marginal branch and remained free of additional stenosis (Figure 1).

Both internal thoracic arteries were harvested in a skeletonized fashion to maximize conduit length and to mitigate the risk of deep sternal wound infection associated with bilateral internal thoracic artery use [2,3,4]. Cardiopulmonary bypass (CPB) was initiated, and cold blood cardioplegia was administered. The aneurysmal ascending aorta was replaced with a Dacron graft. Coronary revascularization was then initiated (Video S1). To access the circumflex artery, the heart was gently elevated using a pad placed under the left ventricle, and the pulmonary trunk was retracted medially with a stay suture. The proximal LAD and the emergence of the left atrial appendage served as key anatomical landmarks. Using low-voltage electrocautery, the overlying fat tissue was carefully incised. Subtle, current-free movements with the cautery blade allowed for the tactile identification of the circumflex artery, distinguished by its unique wall consistency. After confirmation, the artery was meticulously dissected and encircled with a vascular loop, a step facilitated by its superficial location not embedded in the myocardium in this segment. This approach provided optimal visualization and ease of handling of the artery for subsequent anastomosis.

**Figure 2:**
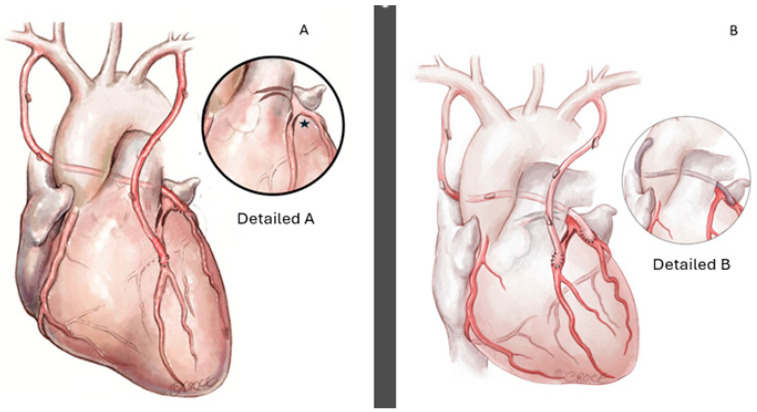
(**A**): Schematic representation of total arterial revascularization with the right internal thoracic artery (RITA) anastomosed to the proximal circumflex trunk and the left internal thoracic artery (LITA) to the left anterior descending artery (LAD). **Detailed A:** 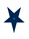 Magnified view of the short, relatively superficial proximal circumflex segment suitable for direct exposure and anastomosis. (**B**): Alternative revascularization strategy. **Detailed** **B:** In case a saphenous vein is used: To reduce the risk of graft kinking, the proximal anastomosis should be placed on the anterolateral aspect of the ascending aorta, with retro-aortic routing through the transverse sinus toward the circumflex territory.

The pedicled right internal thoracic artery (RITA) was tunneled through the pericardium, passing above the superior vena cava, carefully avoiding the phrenic nerve, and through the transverse sinus to reach the circumflex trunk. An incision was made in the circumflex artery, and the distal end of the RITA was shortened and beveled to achieve an exact fit. An end-to-side anastomosis was then completed using a running 8-0 polypropylene suture mounted on an EVERPOINT cardiovascular needle (J&J MedTech, Raritan New Jersey, USA) known for its seemingly better penetration and performance [5]. The left internal thoracic artery (LITA) was subsequently anastomosed to the proximal LAD (Figure 2).

**Figure 3:**
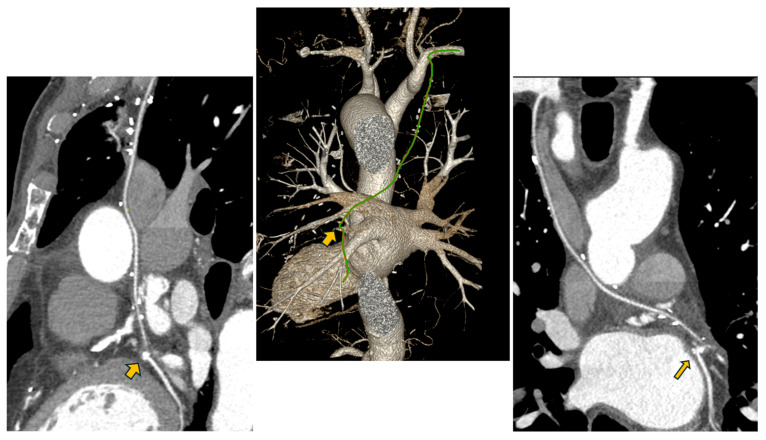
Post-operative computed tomography assessment of the RITA-Circumflex artery direct revascularization of the Circumflex artery one year after surgery. (**Left**): Sagittal thoracic CT slice demonstrating patency of the circumflex coronary artery. (**Middle**): Three-dimensional volume-rendered reconstruction highlighting the retro-aortic course of the RITA, with an unobstructed trajectory, absence of kinking, and the characteristic “cobra head” anastomotic configuration. (**Right**): Retro-aortic pathway of the RITA, anastomosed to the circumflex artery at the site indicated by the arrow.

Following meticulous de-airing of the heart and graft, all clamps were released, and the heart resumed excellent function. CPB was smoothly weaned, and intraoperative graft verification using transit-time flow measurement yielded excellent results. The right internal thoracic graft demonstrated a mean flow of 55 mL/min with a pulsatility index of 1.5, indicating optimal graft performance. The patient’s postoperative recovery was uneventful, and at the 1-year follow-up, he remained in excellent clinical condition, with no complications and no evidence of graft dysfunction, confirmed by a cardiac computed tomography (CT) angiography (Figure 3).

## 3. Discussion

The circumflex territory presents one of the most technically challenging areas to revascularize surgically [6]. Typically, grafts are connected to the marginal branches rather than directly to the circumflex artery itself. This approach often necessitates multiple jump anastomoses, two or three, on small-caliber vessels, frequently requiring intricate angulations. Achieving adequate exposure of these branches can be difficult, especially given the distorted anatomy of a twisted heart and suboptimal exposure. Consequently, the risk of developing anastomotic stenosis is significantly higher compared to anastomoses performed on larger, well-exposed coronary segments.

The present case revisits an older idea using a contemporary arterial strategy. Direct grafting of the main circumflex trunk was already described in the early era of coronary bypass surgery by Cheanvechai and colleagues in 1972, particularly with a venous conduit [7]. Accordingly, the conceptual basis of the present approach is not entirely unprecedented. What distinguishes our report is the use of an in situ RITA, routed through the transverse sinus, to revascularize the proximal circumflex trunk in the setting of modern total arterial revascularization, with both intraoperative flow assessment and 1-year computed tomography confirming excellent conduit function. In that sense, this report should be viewed less as the introduction of a completely novel concept than as a contemporary refinement of a historically recognized but seldom-used anatomical strategy.

From an anatomical standpoint, this strategy is feasible only in selected patients. Although most of the circumflex artery lies deeply within the fat of the atrioventricular groove and may course beneath the coronary sinus, a short proximal segment of approximately 15 mm remains relatively superficial before the vessel dives deeper near the origin of the lateral branches. When present, this segment can be approached surgically using the proximal LAD and left atrial appendage as reliable landmarks. In our patient, this configuration allowed safe encirclement and direct anastomosis (see Video S1). Although exposure of the circumflex trunk requires additional operative time, this may be offset by avoiding the multiple distal anastomoses required with a conventional strategy.

Another important aspect of this technique is the route of the conduit. Prior surgical series have shown that the in situ RITA through the transverse sinus provides a reliable and reproducible route of reaching the circumflex territory, with high early and late patency. Gerola and colleagues reported long-term patency of the RITA through the transverse sinus comparable to that of the LITA and superior to that of saphenous vein grafts, while Ueyama and colleagues demonstrated favorable early results in a large series of posterolateral revascularizations using the same route [8,9]. Sakata and colleagues similarly reported excellent early graft patency with this strategy in a large cohort [10]. These data support the technical soundness of routing the RITA through the transverse sinus in appropriately selected anatomy.

The approach may also offer physiological advantages. By targeting the proximal circumflex trunk, a single proximal inflow source can deliver antegrade perfusion to the downstream circumflex distribution without requiring multiple sequential distal grafts. In addition, using the RITA as an independent in situ graft may reduce some of the flow-distribution concerns associated with composite T- or Y-graft configurations, in which downstream vascular resistance and competitive flow can influence conduit behavior and coronary flow reserve. In selected anatomy, independent inflow to the circumflex territory may therefore simplify graft design while maintaining a physiologically favorable perfusion pattern. This concept is consistent with broader principles of total arterial revascularization, which emphasize durable arterial conduits and conduit configuration tailored to coronary anatomy and target-vessel physiology [11,12,13,14,15].

The value of this technique depends fundamentally on careful patient selection. The most favorable anatomy, based on our view and experience, includes a short but surgically accessible proximal circumflex trunk, a target segment without significant calcification, and distal marginal branches free of diffuse obstructive disease, allowing effective antegrade perfusion from a single proximal anastomosis. Adequate anatomical space for safe exposure and tension-free graft passage through the transverse sinus is likewise essential. Clinically, this strategy appears particularly well suited to situations in which total arterial revascularization is desirable, such as selected left main disease. Conversely, the approach is unlikely to be appropriate when the circumflex trunk is deeply intramyocardial, very short, or heavily calcified, or when extensive distal marginal disease would still necessitate multiple distal grafts. It should therefore be regarded as a selective technical option for suitable anatomy rather than a general solution for all circumflex lesions.

Alternative conduit strategies remain possible. If the RITA is unavailable or unsuitable, a radial artery or saphenous vein graft may theoretically be used to reach the circumflex trunk. It should not, however, be inserted as a side-branch on the LITA but rather on the antero-lateral part of the ascending aorta and brought through the transverse sinus to the circumflex artery, to prevent kinking. (Figure 2B). Our present case, however, supports the idea that the in situ RITA offers a particularly elegant configuration in this setting because it provides independent arterial inflow, favorable geometry, and a direct path through the transverse sinus.

This report has obvious limitations. It describes a single patient and therefore cannot establish superiority over conventional grafting to obtuse marginal branches, sequential grafting, composite arterial configurations, or hybrid revascularization strategies. It also does not address the reproducibility of the exposure in less favorable anatomy. The present case should therefore be interpreted as proof of feasibility rather than as evidence of general applicability. Larger clinical experience will be necessary to determine whether this strategy is consistently reproducible, safe, and durable across a broader patient population.

## 4. Conclusions

Direct revascularization of the proximal circumflex trunk using the in situ right internal thoracic artery routed through the transverse sinus appears technically feasible in carefully selected patients. In the presence of favorable anatomy, this strategy may provide a simple and physiological means of perfusing the circumflex territory while avoiding multiple distal marginal anastomoses. Rather than representing a universal alternative, it should be considered an adjunctive option in selected cases, particularly when total arterial revascularization is desired. Larger clinical experience will be needed to determine its broader applicability and long-term durability.

## Data Availability

The original data presented in the study are included in the article, further inquiries can be directed to the corresponding author.

## Supplementary Materials

The following supporting information can be downloaded at: https://www.mdpi.com/article/10.3390/reports9020134/s1, Video S1: How to achieve an elegant direct circumflex revascularization in isolated left main disease.

## Abbreviations

LAD	Left anterior descending artery
LITA	Left internal thoracic artery
RITA	Right internal thoracic artery
CPB	Cardiopulmonary bypass
